# Single-Anesthesia Event for Lung Nodule Marking and Minimally Invasive Sublobar Resection

**DOI:** 10.3390/jcm14093149

**Published:** 2025-05-01

**Authors:** Noah Gordon, Mae Leef, Richard Irving, Nikolina Madjer, Christopher Bentsen, Daniel Elikman, Alex Cedeno-Rodriguez, Abdul Hamid Alraiyes

**Affiliations:** 1 Advocate Lutheran General Internal Medicine, Park Ridge, IL 60068, USA; noah.gordon2@aah.org (N.G.); mae.leef@aah.org (M.L.); richard.irving@aah.org (R.I.); nikolina.madjer@aah.org (N.M.); christopher.bentsen@aah.org (C.B.); 2 Department of Kinesiology, Nutrition, and Health, Miami University Oxford Campus, Oxford, OH 45056, USA; danikme77@gmail.com; 3 Centro Cardiovascular de Puerto Rico y el Caribe Thoracic Surgery, San Juan 00936, Puerto Rico; alexr.cedeno@gmail.com; 4 Advocate Lutheran General Interventional Pulmonology, Park Ridge, IL 60068, USA

**Keywords:** interventional pulmonology, lung nodule, lung cancer, diagnosis, sublobar resection, bronchoscopy, marking, retrospective, anesthesia

## Abstract

**Background:** Non-small cell lung cancer (NSCLC) accounts for a significant number of new lung cancer diagnoses each year, which, if identified early, may be surgically removed with curative intent. It is also the most common indication for a sublobar resection due to its equal efficacy in carefully selected patients. From the time of diagnosis to surgery, however, traditionally, there are three separate anesthesia-dependent events: (1) diagnostic bronchoscopy plus lymph node staging, (2) lung nodule marking, and (3) surgical resection. This study evaluated the viability of performing a pulmonary nodule marking and sublobar resection under a single-anesthesia-dependent event at a large community hospital. **Methods:** The study group was a single-center retrospective cohort of patients, scheduled for same-day marking and sublobar resection and admitted to a large community hospital between 6 January 2023 and 23 May 2023. Prior to arrival, patients had received cardiac surgical clearance, pulmonary function testing, and positron emission tomography to ensure their appropriateness for surgical intervention. Data regarding procedural time, anesthesia time, and hospital length of stay was collected retroactively though the electronic medical record. **Results:** A total of 12 patients with 16 pulmonary nodules were included. Results demonstrated a mean turnover time of 33 min between completing pulmonary fiducial marking and starting the sublobar resection. The estimated mean total time saved was 231 min. The average hospital length of stay was 1.83 days. **Conclusions:** Combining pulmonary fiducial marking and sublobar resection within a single-anesthesia-dependent event offers an opportunity to decrease total perioperative time and the time from diagnosis to curative intervention.

## 1. Introduction

A sublobar resection is the removal of a portion of a lobe of the lung rather than an entire lobe, known as a lobectomy. There are two main types of sublobar resections, namely wedge resection and segmentectomy. A wedge resection removes the least amount of lung tissue but risks excluding surrounding lymph nodes; therefore, segmentectomy, which removes an entire segment of lung, is preferred for a decreased likelihood of regional oncologic recurrence [1].

Before proceeding with sublobar resection, a comprehensive presurgical workup is essential to ensure that patients are appropriately selected for this lung-sparing approach. High-resolution computed tomography (CT) scans with volumetric analysis are used to assess the tumor’s size, location, and relation to vital structures, while positron emission tomography (PET)-CT scans help evaluate any evidence of regional or distant metastasis. Notably, prior to standard use of the PET scan pre-operatively, about 30 percent of patients with NSCLC had metastasis found within 12 months following surgery [2]. In cases where lymph node involvement is a concern, endobronchial ultrasound (EBUS) is primarily utilized for the assessment of lymph nodes through EBUS-guided transbronchial needle aspiration (EBUS-TBNA) as part of echoendoscopic mediastinal staging. Robotic assistance is not required to perform EBUS-TBNA. Additionally, bronchoscopy with transbronchial needle aspiration (TBNA) or transbronchial biopsy is commonly used to obtain a diagnosis of pulmonary nodules. While various tools and guidance systems—such as fluoroscopy or radial EBUS—can assist in achieving this diagnosis, robotic bronchoscopy is not always available or necessary. If mediastinal lymph nodes are found to be involved, patients may no longer be suitable for sublobar resection and may require either lobectomy or nonsurgical treatment options [3]. Pulmonary function testing (PFT) is also a crucial component of preoperative evaluation, as it helps predict postoperative lung function and determine whether the patient will tolerate the planned resection. Those with borderline pulmonary reserve may benefit the most from sublobar resection due to its lung-preserving advantage [4]. Given that many patients with lung cancer have coexisting cardiovascular disease, preoperative cardiac clearance, including echocardiography and stress testing, is often required to assess perioperative risk and optimize patient outcomes.

As technology advances, particularly with the development of robotic bronchoscopy and real-time 3D imaging, the field of pulmonary diagnostics has seen significant improvements in the ability to detect and biopsy small pulmonary nodules. The integration of robotic-assisted navigation with high-resolution imaging has allowed for unprecedented precision in accessing and sampling peripheral lung nodules, even those measuring less than 20 mm [5]. Historically, nodules of this size and location posed a significant challenge for tissue sampling due to their inaccessibility and the limitations of traditional bronchoscopy or CT-guided percutaneous biopsy. However, with robotic-assisted bronchoscopy, pulmonologists can now navigate the complex architecture of the bronchial tree with greater accuracy, improving the diagnostic yield of biopsies while reducing complications such as pneumothorax and hemorrhage [6,7]. This has led to an increase in the detection of small, early-stage lung cancers, particularly non-small cell lung cancer (NSCLC) measuring 20 mm or less [5,8].

With the ability to detect malignancies at an earlier stage, there has been a notable shift in surgical management strategies, particularly in the increasing utilization of sublobar resection as an alternative to traditional lobectomy. Historically, lobectomy has been considered the gold standard for resectable NSCLC, as it has been associated with lower recurrence rates and improved long-term survival [9]. However, recent clinical trials have demonstrated that for carefully selected patients with small, node-negative peripheral NSCLC, sublobar resection, which includes segmentectomy or wedge resection, is noninferior to lobectomy in terms of disease-free survival and overall survival [10,11]. Specifically, the Cancer and Leukemia Group B (CALGB) trial found no significant difference between the two approaches in recurrence rates or long-term survival, with five-year disease-free survival at 63.6 percent for sublobar resection and 64.1 percent for lobectomy and five-year overall survival at 80.3 percent and 78.9 percent, respectively [10]. This shift toward lung-sparing surgical strategies has been driven by multiple factors, including earlier diagnosis facilitated by advanced imaging and bronchoscopy techniques, improvements in intraoperative lymph node staging to confirm node-negative disease, and a growing recognition of the benefits of preserving pulmonary function. Preserving pulmonary function is particularly relevant for patients with preexisting lung conditions such as chronic obstructive pulmonary disease (COPD) or interstitial lung disease (ILD), who may not tolerate the functional loss associated with lobectomy.

As the landscape of early-stage lung cancer management continues to evolve, the increasing use of sublobar resection for small, node-negative NSCLC represents a significant shift in surgical oncology. The ability to detect and diagnose lung cancer at earlier stages through robotic-assisted bronchoscopy has played a major role in this transition, allowing for a more personalized and less invasive approach to treatment. Ongoing research and advancements in molecular tumor profiling, artificial intelligence-driven imaging analysis, and minimally invasive surgical techniques will likely further refine patient selection and enhance treatment outcomes. By integrating these emerging technologies, the field of thoracic surgery is moving toward a more individualized and function-preserving approach to lung cancer management, ultimately improving both survival and quality of life for patients diagnosed at an early stage [1].

Integrating interventional pulmonology and thoracic surgery within the same section allows for a more streamlined approach to diagnosing and treating NSCLC nodules. This structure also enables a highly coordinated workflow where biopsy, localization, and surgical resection can be performed within the same anesthesia event.

A major advantage of this model is the ability to facilitate seamless collaboration between specialists, ensuring that patients progress smoothly from diagnostic evaluation to therapeutic intervention. When a lung nodule is detected, a structured process is initiated, incorporating advanced imaging, robotic-assisted bronchoscopy for biopsy and staging, and multidisciplinary case review. If the nodule is confirmed as malignant and deemed suitable for surgical resection, the same procedural setting allows for intraoperative localization and immediate resection by the thoracic surgery team. This approach minimizes the need for multiple hospital visits, reduces anesthesia exposure, and shortens the interval between diagnosis and treatment, leading to more efficient patient care.

Intraoperative localization is enhanced with the use of fiducial markers—a small object placed within tissue to serve as a recognizable reference point for surgeons. The primary goal of fiducial markers is to minimize the loss of normal lung parenchyma while still ensuring adequate surgical margins. Nodules that are less than 2 cm in size or ground-glass are particularly challenging for surgeons to localize due to being more difficult to palpate intraoperatively. Therefore, in these cases, fiducial markers are routinely implemented. One notable limitation, however, is that fiducial marking for nodules located further than 2 cm from the pleural surface may not easily be detected and is therefore less effective in these situations.

The ability to track the progression of NSCLC nodules from detection to treatment within a single coordinated event enhances patient management and strengthens collaboration between interventional pulmonologists and thoracic surgeons. By integrating these services into a unified model, this approach not only optimizes resources and procedural efficiency but also enhances the overall patient experience by providing a more rapid, multidisciplinary, and well-coordinated approach to lung cancer management.

This study aimed to assess the feasibility of utilizing robotic-assisted bronchoscopy for the endoscopic marking of target lung nodules in conjunction with sublobar resection within a single-anesthesia event. By integrating these procedures into a single coordinated intervention, the goal was to enhance surgical precision while minimizing procedural delays. The hypothesis was that this combined approach would lead to a reduction in hospital length of stay by streamlining the transition from diagnosis to treatment, thereby decreasing the overall time between nodule detection, localization, and definitive resection. Additionally, the study sought to evaluate whether this strategy could optimize resource utilization, improve patient outcomes, and reduce the need for multiple hospital visits, ultimately leading to a more efficient and patient-centered approach to early-stage lung cancer management.

## 2. Materials and Methods

This is a single-center, observational, retrospective cohort study that received a human subject research waiver approval from the Institutional Review Board (IRB). The study examined patients with non-small cell lung cancer (NSCLC) who had pulmonary nodules less than 2 cm in size and underwent lung nodule marking followed by sublobar resection during the same anesthesia-dependent event between 6 January 2023, and 23 May 2023. All patients provided informed consent for both procedures prior to scheduling.

### 2.1. Patient Selection

Patient selection criteria included a previously confirmed diagnosis and accurate staging based on pathology reports obtained from a prior combined procedure for lung nodule biopsy and lymph node staging. Eligible patients had a <2 cm peripherally located lung nodule and had completed preoperative evaluation with pulmonary function testing, PET-CT imaging, and cardiac risk assessment for surgical clearance. The diagnostic and staging procedure was performed using robotic-assisted bronchoscopy (RAB), with the final diagnosis and staging confirmed within five days following the procedure. To minimize the need for an additional anesthesia event solely for lung nodule marking, a combined approach was planned, allowing lung nodule marking and surgical resection to occur in a single operative session once all prior evaluations were completed and confirmed. Likewise, the performance of single-event anesthesia required close coordination between the interventional pulmonary and thoracic surgery teams. As an attempt to limit external delays to the cases, patients were scheduled to be the first case of the day in a majority of the cases.

The study initially included 14 patients. Of this cohort, 2 patients were excluded from the analyzed data. One was excluded due to a change in operative plan to a lobectomy. The other patient was excluded for incomplete data collection intraoperatively.

### 2.2. Data Collection

Data were retrospectively collected and exported from the electronic medical record (EMR) and included the following: anesthesia time, procedural time, turnover time between each procedure, total operating room time, and time from hospital admission to transfer out of the recovery area. Anesthesia and procedure time were derived from the manually recorded time from start to end. Operating room time started when the patient entered the operating room until they moved to the post-op recovery area. The turnover time was the time spent transitioning from marking to thoracic surgical intervention. Recovery time included all of the time spent in recovery.

### 2.3. Surgical Technique

**Figure 1:**
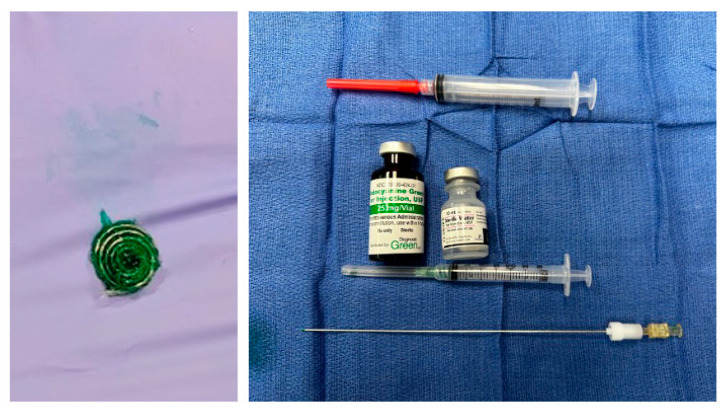
Showing the marking coil on the left and the indocyanine green (ICG) loading kit on the right.

On the day of the procedure, the operating room is fully prepared in advance to accommodate both the Ion™ Endoluminal System (Sunnyvale, CA, USA) (Intuitive’s robotic-assisted bronchoscopy platform for minimally invasive lung biopsy) and the Da Vinci^®^ Surgical System (Intuitive’s robotic-assisted thoracic surgery platform), ensuring a seamless transition between the diagnostic and surgical phases. Prior to the patient’s arrival, a multidisciplinary team—including interventional pulmonology, thoracic surgery, anesthesia, and radiology—reviews the preoperative planning CT scan, during which the target lung nodule is carefully mapped and labeled for precise bronchoscopic navigation. Following induction of general anesthesia, the procedure begins with RAB, utilizing the pre-planned navigation pathway to guide the bronchoscope to the target lesion. Once the area is reached, a cone-beam CT (CBCT) spin is performed to confirm accurate localization of the nodule in real time. This imaging ensures that the navigational alignment is precise and that the bronchoscope is properly positioned at the intended site. After confirming the location, the nodule is digitally segmented on the cone-beam CT, and the lesion is then augmented onto live fluoroscopy, creating a dynamic overlay that enhances real-time visualization. This combination of RAB and augmented fluoroscopy allows high-fidelity targeting and confident confirmation of the nodule’s position, which is essential for successful localization and resection. Robotic-assisted bronchoscopy is used to navigate to the lung nodule following the pre-planned navigation pathway. Once the lung nodule is reached by the robotic bronchoscope and confirmed with augmented imaging and fluoroscopy, marking is performed through the bronchoscopy catheter using two techniques: through direct injection of indocyanine green (ICG) into the lesion using the Ion Flexision™ needle, followed by deploying an ICG-soaked coil (Cook Tornado, 3 × 7 mm, 0.035 inch) at the site with the Medtronic superDimension^TM^ (Minneapolis, MN, USA) marker delivery kit (Figure 1).

**Figure 2:**
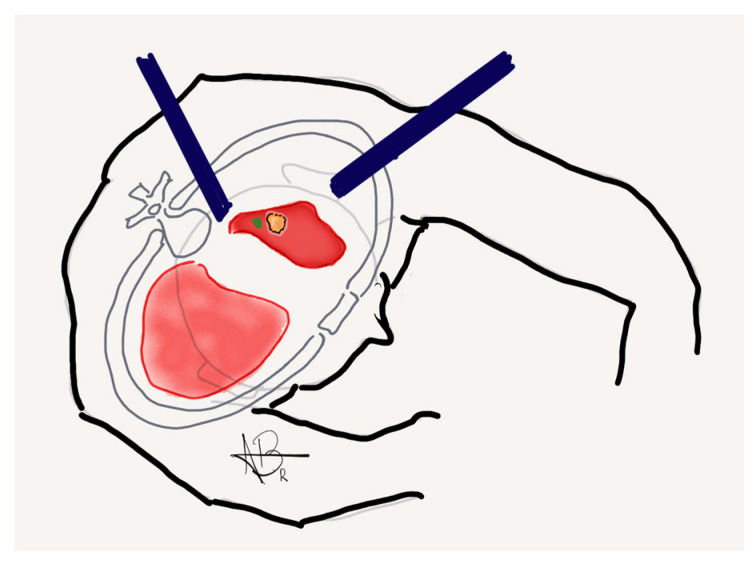
Illustrative graphic depicting a lung nodule (highlighted in yellow) and the localization marker (highlighted in green), demonstrating the spatial relationship between the lesion and the pleural surface for surgical resection.

Key technical considerations include ensuring that the marking is placed close to the pleural surface, ideally within the area corresponding to the intended surgical resection line, to allow the thoracic surgeon to perform a wedge resection with clear margins. Moreover, the marker should be no more than 1 cm from the visceral pleura, as this proximity is necessary for the ICG signal to be reliably visualized during the intraoperative robotic surgical portion (Figure 2) [12].

**Figure 3:**
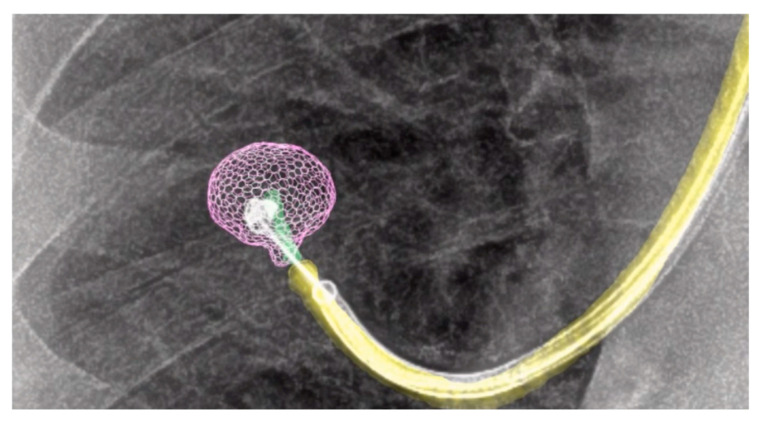
Illustrative fluorosocopy findings of the augmented lung nodule in pink and the augmented bronchoscope in yellow, with the life deployment of the marking coil into the lung nodule under live fluoroscopy.

After successful localization of the lung nodule using robotic-assisted bronchoscopy (RAB) in conjunction with cone-beam CT (CBCT) and augmented fluoroscopy, nodule marking is performed with precision under real-time imaging guidance. The integration of these technologies ensures that the bronchoscope is accurately positioned at the target lesion, allowing for precise deployment of the chosen localization method—whether via the direct injection of indocyanine green (ICG) or the placement of an ICG-soaked coil. This step is critical to enable accurate intraoperative identification and resection of the nodule during the subsequent surgical procedure (Figure 3).

**Figure 4:**
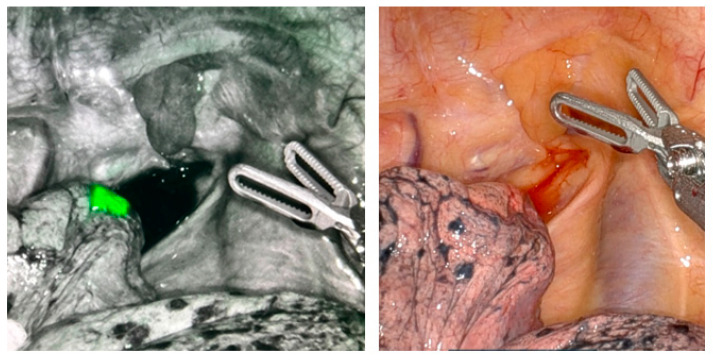
Intraoperative localization of a lung nodule using near-infrared fluorescence imaging with indocyanine green (ICG). (**Left**): Firefly™ near-infrared mode reveals a bright green, fluorescent signal at the site of the ICG-marked lung nodule, enabling precise intraoperative identification. (**Right**): standard white-light view of the same anatomical region without fluorescence.

Following successful lung nodule marking, the robotic bronchoscope is carefully withdrawn, and the procedure transitions to the surgical phase. The patient is repositioned and placed on single-lung ventilation in preparation for robotic-assisted thoracic surgery. With the nodule already marked, the next critical step involves localizing the lesion intraoperatively using Firefly™ (Sunnyvale, CA, USA) near-infrared fluorescence imaging, which detects the presence of indocyanine green (ICG). Under this specialized light, the ICG fluoresces bright green, making the marked lesion clearly visible and easily distinguishable from the surrounding lung parenchyma (Figure 4). This precise visualization significantly reduces the time required to identify the target lesion during surgery and enables the thoracic surgeon to perform a targeted wedge resection with clear margins. Additionally, accurate localization allows for minimally invasive resection, preserving a greater volume of healthy lung tissue and supporting better postoperative outcomes.

**Figure 5:**
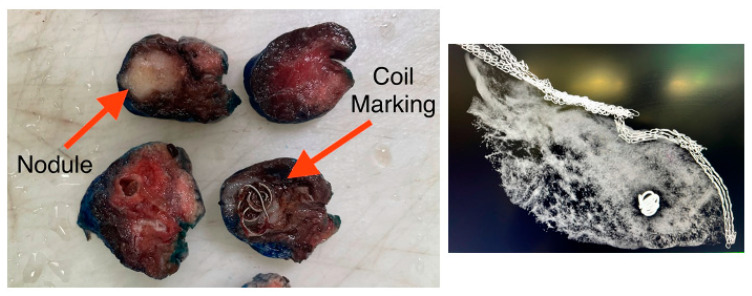
Confirmation of complete lung nodule resection using fluoroscopy and gross pathology. (**Left**): gross pathological specimen showing the resected lung nodule and the indocyanine green (ICG)-soaked coil used for intraoperative localization. The nodule (labeled) is fully contained within the resection margins, with the coil marker embedded adjacent to the lesion, confirming accurate targeting. (**Right**): intraoperative fluoroscopic image of the resected specimen displaying the coil marker within the tissue, verifying successful localization and resection of the intended lesion.

Resection of the lung lesion is confirmed intraoperatively through fluoroscopic evaluation, which verifies that the fiducial marker—previously placed during robotic bronchoscopy—is contained entirely within the resected specimen, ensuring that the targeted nodule has been accurately excised. This radiographic confirmation provides real-time assurance that the lesion has been completely removed with an appropriate orientation. Following resection, a pathological assessment further validates the completeness of the procedure, confirming that the tumor is entirely encompassed within the surgical margins, with no microscopic residual disease. Dual-modality verification—using both intraoperative fluoroscopy and histopathologic evaluation—ensures complete oncologic resection with negative margins, supporting the success of the combined bronchoscopic and surgical approach (Figure 5).

In our study, indocyanine green (ICG)-soaked coils were used for lung nodule localization with the specific aim of performing both localization and surgical resection under a single-anesthesia event. We appreciate the important contribution OF Bawaadam et al., who demonstrated the feasibility of using ICG-soaked coils for delayed surgical resections—showing that the fluorescent signal can remain visible for up to 9 days post placement [12,13]. Their work highlights the flexibility and durability of this technique. Building on this concept, our approach was designed to minimize the number of anesthesia-dependent procedures by consolidating bronchoscopic localization and robotic resection into the same operative session. The ICG-soaked coils were bronchoscopically deployed into or adjacent to the target lung nodule, providing a bright green, fluorescent signal under near-infrared imaging during surgery. This allowed accurate, real-time identification of the lesion and facilitated precise wedge resection with clear margins. In addition to their fluorescent properties, the coils were palpable with robotic graspers and easily visualized on intraoperative fluoroscopy and ultrasound, offering multiple layers of confirmation. This strategy supported lung-sparing resections while reducing perioperative risk and improving workflow efficiency for both patients and providers.

## 3. Results

There were 12 cases for which a total of 16 nodules were procedurally marked and removed through sublobar resection. Patient demographics and nodule characteristics are illustrated in Table 1.

**Figure 6:**
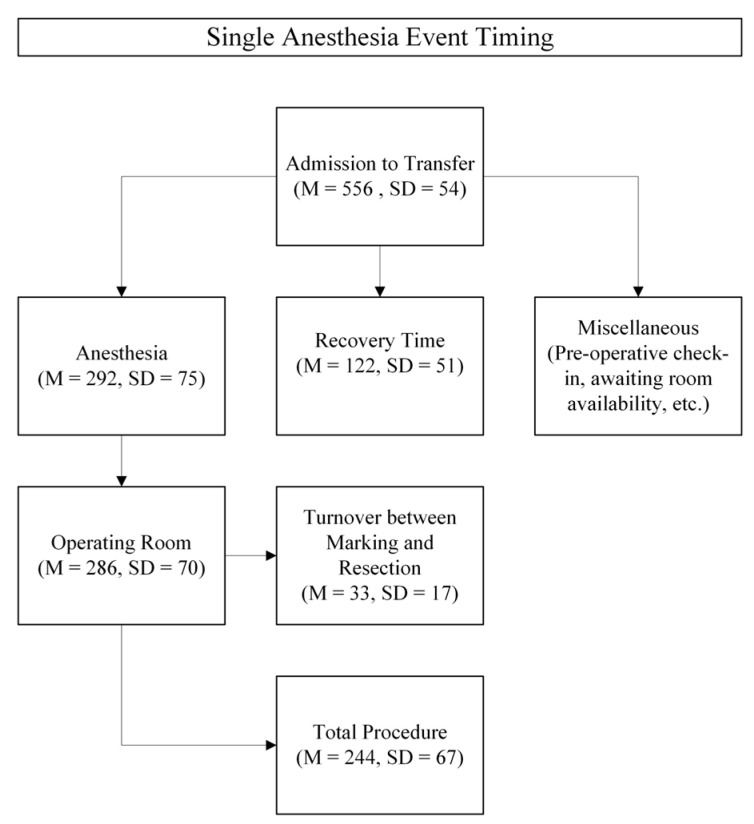
Mean (M) time and standard deviation (SD) represented in minutes spent per case, divided into phases. Time was recorded from the time of patient admission to transfer out of recovery.

As represented in Figure 6, the utilization of time was recorded, averaged, and categorized into the overarching task. The time-related benefits of combining the two procedures include not having to repeat recovery time, which took a mean of 122 min, and miscellaneous time, which, although not measured, can be estimated to be about 142 min. This calculated mean is based on subtracting the mean “admission to transfer” time from the “anesthesia” plus “recovery” time. On the contrary, the time cost of performing the two procedures together is best represented as the “turnover time between marking and resection” with a mean of 33 min and standard deviation of 17 min. In total, combining the procedures provided a net benefit of approximately 231 min. Notably, the turnover time number decreases immensely if the first attempt at combining the two events is not included, as it took 82 min. Turnover time improves to a mean of 28 min with a standard deviation of 8 min when removing this attempt, which was slowed by the lack of experience amongst supporting staff during the change in the procedural process. Therefore, this total time benefit likely underestimates the true benefit conferred.

**Figure 7:**
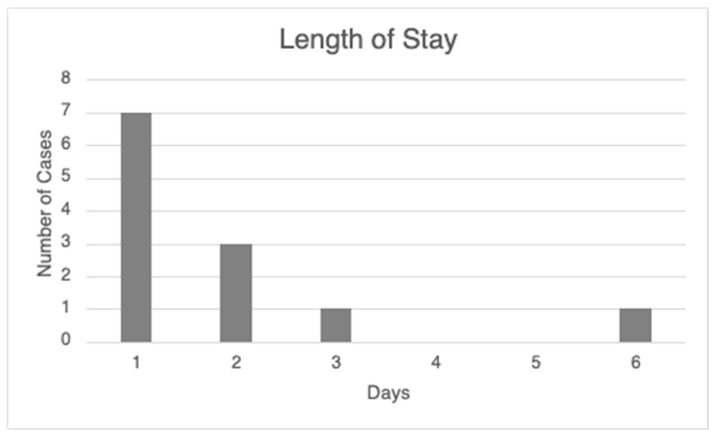
Hospital admission length of stay in days for each of the 12 included patient cases.

The median post-surgical hospital length of stay (LOS) was 1 day, and the mean was 1.83 days, as depicted in Figure 7, with a graphically depicted positive skew. Notably, the case associated with a 6-day LOS was complicated by an air leak following wedge resection, leading to the prolonged hospital course. As this complication is uncommon, the median serves as a more accurate indicator of our expected hospital course for single-anesthesia-dependent marking and sublobar resection.

Among the 12 patients included in the study, only one experienced a postoperative complication, consisting of a prolonged air leak that resolved by postoperative day six. All patients were monitored postoperatively through regular thoracic surgery clinic visits. No additional postoperative complications were observed. Surveillance with chest CT imaging and clinical follow-up continued for all patients, with no evidence of recurrence or new complications to date.

## 4. Discussion

According to the National Cancer Institute, lung cancer remains the leading cause of cancer-related deaths in both men and women, accounting for more fatalities each year than breast, prostate, and colorectal cancers combined [14]. Despite advancements in treatment, lung cancer often goes undetected until it reaches advanced stages—making early detection critical for improving survival outcomes. This underscores the urgent need for continued innovation in screening, diagnosis, and minimally invasive procedures that can identify and treat lung cancer in its earliest, most treatable stages [14]. The prognosis of lung cancer is influenced by several key pathological markers that reflect the tumor’s biological behavior and potential for progression. Cell type plays a critical role—non-small cell lung cancer (NSCLC) generally has a better prognosis than small cell lung cancer (SCLC), which is more aggressive and prone to early metastasis. The presence of lymphatic and vascular invasion is associated with a higher risk of regional and distant spread, indicating a more advanced and potentially less favorable outcome. Tumor size is another important factor, as larger tumors are more likely to invade surrounding structures and metastasize. The five-year survival rate for lung cancer has been approximately 15% for three decades [15]. Stage and treatment approach are the largest factors affecting survival [15]. Therefore, we hypothesized that a more streamlined approach from detection to resection may offer several benefits, including a potential mortality benefit.

Formerly, lobar resection has been the standard of care for patients with early-stage non-small cell lung cancer (NSCLC), following a landmark 1995 clinical trial that demonstrated a survival advantage over sublobar resection [9]. However, recent high-quality evidence has redefined this paradigm. In 2023, Altorki et al. published a pivotal randomized controlled trial evaluating sublobar versus lobar resection in patients with T1aN0 NSCLC, defined as tumors 2 cm or less in size with no nodal involvement. In this study, node-negative status was confirmed intraoperatively, and patients were randomized to receive either lobectomy or sublobar resection. The results showed that sublobar resection was not inferior to lobectomy with respect to disease-free survival, while also offering the added benefits of preserving lung parenchyma and maintaining pulmonary function [10].

In our study, we fully implemented the surgical and oncologic criteria outlined in the Altorki et al. trial, including preoperative and intraoperative staging, tumor size selection (≤2 cm), and confirmation of nodal negativity prior to resection. As a result, sublobar resection was selected as the definitive treatment approach for our patients, aligning with contemporary evidence while supporting lung-sparing, curative-intent surgery. This approach allowed us to optimize oncologic outcomes while minimizing functional impairment in patients with early-stage NSCLC.

Historically, the pathway from the initial discovery of a suspicious lung nodule to definitive diagnosis and surgical resection has been lengthy and fragmented; for instance, one analysis reported that the median time from radiographic identification of a pulmonary nodule to therapeutic surgical resection was approximately 98 days [16]. Delays in the management of suspicious lung nodules are often multifactorial. Once a concerning lesion is identified, the diagnostic and staging process involves multiple steps, beginning with advanced imaging, such as high-resolution CT and PET scans, to further characterize the lesion and assess for metastatic disease. This is followed by tissue acquisition, which may include transthoracic or transbronchial biopsy of the nodule itself, as well as endobronchial ultrasound (EBUS) for mediastinal and hilar lymph node staging. If malignancy is confirmed or strongly suspected and the patient is considered a surgical candidate, further evaluation is required to assess operative risk, including cardiac risk stratification, pulmonary function testing, and sometimes additional consultations. Each of these steps requires separate appointments, contributing to delays—especially in the context of scheduling constraints, preoperative wait times, and patient availability or preferences. Even after surgical clearance is obtained, patients frequently undergo robotic bronchoscopy with fiducial marker placement or dye localization to guide intraoperative identification of the lesion—yet another anesthesia-dependent procedure that is typically scheduled separately from the resection. This drawn-out process can lead to delayed treatment, disease progression, and heightened patient anxiety. It also creates inefficiencies in healthcare delivery, particularly in systems where multidisciplinary coordination is lacking or siloed. These challenges underscore the need for integrated care models and advanced technologies that can streamline diagnosis and expedite time to treatment—especially for early-stage lung cancer, where timely intervention is critical for curative outcomes.

Our approach to solitary lung nodules—from discovery to diagnosis and treatment—has been significantly expedited by the integration of Interventional Pulmonology and Thoracic Surgery within a single division. This unified structure fosters a daily multidisciplinary workflow, allowing for immediate review and planning for all new lung nodules, whether identified incidentally on imaging or through our dedicated lung cancer screening program. Once a nodule is detected, a coordinated plan is rapidly initiated for clinical evaluation and biopsy, streamlining the patient journey and reducing delays in care. The incorporation of robotic-assisted bronchoscopy coupled with real-time cone-beam CT imaging has dramatically enhanced our diagnostic capabilities. These technologies enable precise navigation and sampling of nodules as small as 5 mm and up to 20 mm, many of which were previously considered too small or inaccessible for reliable biopsy. As a result, we have seen a substantial improvement in the diagnostic yield of early-stage lung cancers, particularly Stage I and II non-small cell lung cancer (NSCLC), and specifically in nodules under 2 cm [5]. What truly differentiates our approach is the high level of collaboration and scheduling synchrony between the Interventional Pulmonology and Thoracic Surgery teams. This enables us to perform both lung nodule localization and sublobar robotic resection during a single-anesthesia event, minimizing procedural burden on the patient while optimizing efficiency and outcomes. This integrated, patient-centered model has significantly shortened the time from nodule detection to definitive therapy—ensuring timely intervention, reducing anxiety for patients, and improving the likelihood of curative treatment.

Combining robotic lung nodule localization and surgical resection into a single procedural event offers significant clinical and patient-centered advantages. This streamlined approach can accelerate the time to treatment, enabling earlier intervention—which may not only improve survival outcomes but also reduce the emotional burden associated with prolonged diagnostic and treatment timelines. By minimizing the number of separate procedures, patients face fewer hospital visits, shorter overall recovery time, and less disruption to their daily lives. Importantly, consolidating these procedures reduces exposure to general anesthesia, which carries inherent risks such as hemodynamic instability, airway injury, aspiration, and postoperative infection. By decreasing the number of anesthesia-dependent events, we are therefore decreasing cumulative risk. This can be particularly beneficial in patients with comorbidities that place them at a higher risk of complications while undergoing general anesthesia. In addition, performing robotic-assisted nodule marking and sublobar resection in the same session enhances surgical precision. It allows for more accurate localization and targeted excision of malignant tissue, helping to preserve healthy lung parenchyma. As a result, postoperative recovery is often smoother, and hospital length of stay is significantly reduced, contributing to both improved patient outcomes and more efficient resource utilization.

Two studies similarly investigated the benefits of a combined single-anesthesia event for marking and resection in NSCLC. The first study included 40 patients in the single-anesthesia robotic navigational bronchoscopy with biopsy and resection (SABR) group and 30 patients in a staggered approach group. They found that the SABR approach offered a significant reduction in the time from diagnosis to treatment and reduced the cost of care [17]. Another study by Fujiwara-Kuroda et al. investigated the feasibility of performing this combined approach. They noted the disadvantages of this method, including longer operating time, limited patient positioning, and the need for a hybrid operating room [18]. However, overall, they concluded that this could be a simple, useful, and practical method.

While this integrated, single-anesthesia approach to lung nodule localization and resection demonstrates considerable promise, several important limitations and logistical challenges must be acknowledged. One of the primary concerns is the prolonged total procedural time, which includes not only the robotic bronchoscopy and surgical resection components but also the turnover time between these phases within the operating room. This additional time under general anesthesia may be clinically significant, particularly in patients with multiple comorbidities or limited physiologic reserve.

The suitability of this combined approach is highly dependent on specific clinical variables, including the size, depth, and location of the pulmonary nodule, as well as the patient’s overall performance status. Certain nodules may be technically challenging to reach or mark bronchoscopically, making them less ideal candidates for this protocol. Moreover, successful implementation requires robust multidisciplinary collaboration, involving thoracic surgery, interventional pulmonology, anesthesia, radiology, oncology, and the patient. Pre-procedural planning and real-time intraoperative coordination are critical, and this level of integration may not be readily available at all institutions.

Looking ahead, prospective studies are needed to evaluate the safety and effectiveness of this approach compared to the traditional, staged method involving multiple anesthesia events. Key outcome measures should include not only morbidity and mortality, but also procedure-related complications, length of hospital stay, time to therapy, patient satisfaction, and long-term oncologic outcomes. Additionally, qualitative research incorporating patient-reported experiences and preferences will be essential for assessing the broader value of this model of care. A comprehensive understanding of these clinical and operational dynamics will be crucial in determining whether this streamlined, single-anesthesia pathway can be widely adopted as a new standard in the multidisciplinary management of early-stage lung cancer.

## 5. Conclusions

Lung cancer remains the leading cause of cancer-related death and the current model of detection and treatment is inefficient. Although some delay from time of diagnosis to treatment is necessary to ensure surgical viability, the time between lung fiducial marking and sublobar resection can be improved by combing the procedures to occur within a single-anesthesia-dependent event. In the future, prospective studies with a large and diverse patient cohort are needed to better understand the safety and effectiveness of this approach compared to the traditional, staged model of multiple anesthesia events.

## Figures and Tables

**Table 1:** Patient demographics including sex and age. Pulmonary nodule characteristics including size, location, density, and final diagnosis. Sublobar resection technique utilized and observed complications.

Characteristics	*n* (%)
Total Patients	12 (85.71)
Male	10 (83.33)
Female	2 (16.66)
Age (years)	
Mean	69.42 ± 9.10
Median	68.5
Range	53–83
Nodule Size (mm) ^1^	
Mean	17.38 ± 4.90
Median	18.50
Range	8–26
Nodule Locations	
Right upper lobe	6 (37.50)
Right middle lobe	1 (6.25)
Right lower lobe	6 (37.50)
Left upper lobe	3 (18.75)
Left lower lobe	0 (0.00)
Nodule Density	
Solid	10 (62.50)
Part-solid	4 (25.00)
Ground-glass	2 (12.50)
Sublobar Resection Strategy	
Segmentectomy	7 (43.75)
Wedge Resection	9 (56.25)
Final Diagnosis	
Adenocarcinoma	11 (68.75)
Squamous Cell	1 (6.25)
Typical Carcinoid	1 (6.25)
Non-Pulmonary Metastasis ^2^	3 (18.75)
Post-Operative Complication	
No complications	11 (91.67)
Prolonged air leak	1 (8.33)

^1^ Nodule size reported is the size determined by pathology measurement following surgical resection. ^2^ Final diagnoses as determined by pathology of surgically resected tissue. These pulmonary nodules were confirmed to be adenocarcinoma on robotic-assisted bronchoscopy (RAB) biopsy and on a surgical specimen found to be inconsistent with pulmonary etiology.

## Data Availability

The raw data supporting the conclusions of this article will be made available by the authors on request.
